# Combined Approach of Ligand Targeted and Stimuli-triggered Nanocarriers: a State-of-the-art Strategy for Cancer Treatment

**Published:** 2017

**Authors:** Azadeh Haeri


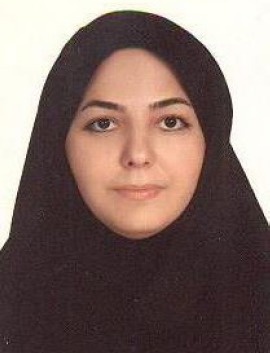


Cancer is one of the world´s most devastating diseases and chemotherapy remains one of the basic treatment methods. In spite of high cytotoxicity of chemotherapeutics against tumor cells, efficacy is greatly hampered by extensive distribution throughout the body following systemic administration, lack of tumor-specific localization and activity, and rapid drug clearance from circulation. These issues are responsible for drug-induced toxicity in healthy labile tissues and organs, serious side effects, and suboptimal drug levels in tumor sites that eventually limit drug dosing and anti-tumor efficacy (1).

Drug encapsulation in biocompatible nanocarriers, nanotherapeutics, that can overcome biological barriers and reach diseased tissues offers a promising approach to lower systemic exposure and toxicity, improve tumor-specific delivery, and increase therapeutic index. Nanotherapeutics offer the possibility to encapsulate hydrophilic and hydrophobic drugs, protect therapeutic molecules, extend their blood circulation, modify their biodistribution, and facilitate the combination regimens by co-encapsulating multiple active ingredients. Various studies have demonstrated that well-formulated nanoparticles show prolonged circulation time and preferentially accumulate in tumors site via the enhanced permeability and retention (EPR) phenomenon (so-called "passive targeting") (2).

However, tumor accumulation and cancer cell bioavailability of chemotherapeutics relying on conventional and stealth (PEGylated) nanocarriers by passive targeting seem still far from optimal to guarantee improved therapeutic efficacy in clinical practice. Specific nanoparticle accumulation in tumors and promoting drug release into the specific site remain major challenges that should be addressed.

Ligand conjugated nanoparticles are the well-studied approach to target cancer cells and maximize nanoparticles accumulation at tumor sites. Cancer results in overexpression of many cell surface receptors that can be exploited for targeting via specific affinity ligands such as antibodies, antibody fragments, nanobodies, aptamer, folate, transferrin, epidermal growth factor (EGF), and peptides. Active tumor-targeting by ligand functionalized nanocarriers leads to increased accumulation and retention of nanotherapeutics in the tumor vasculature as well as specific and efficient uptake by target cancer cells (3).

**Scheme 1:**
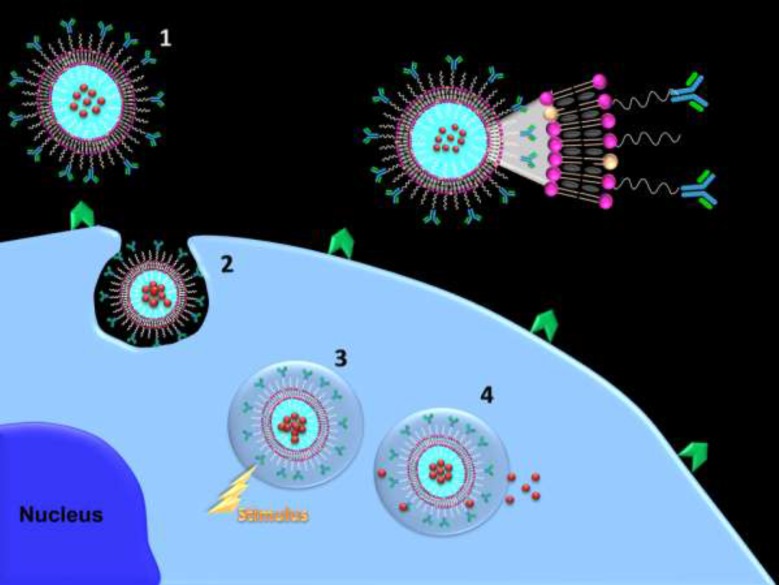
Schematic illustration of combining cancer cell targeting and triggered drug release capabilities

Since chemotherapeutics interaction with their target is required for their efficacy, upon nanoparticles binding to the tumor vasculature or internalization into tumor cells, it would be a promising strategy to actively trigger drug release from the nanoparticle to facilitate intracellular bioavailability and drug-target interaction. 

Stimuli-responsive nanostructured carriers are very attractive since such nanosystems allow on demand, remote, and repeatable switching on or off drug release by an external/internal stimulus. Magnetic field, ultrasound, light, pH, temperature, redox, specific biomolecules, and cellular enzymes are some of stimulus. On demand triggered release nanosystems can control the extent and duration of drug release into tumor cell. Ideally, such carriers entrap and retain the drug with no premature release at undesirable sites in the body and subsequent drug release would be triggered upon stimulation at the action site (4).

The potential of a combination of these two approaches in one targeted triggered nanostructured carrier can enhance therapeutic potential and greatly diminish adverse drug toxicities (scheme 1). Combining cancer cell targeting and triggered drug release capabilities can shift availability of free drug upon triggering from the extracellular to the intracellular compartment while combination of vascular targeting and triggered drug release capabilities can enhance accumulation of nanocarriers in the close proximity of target site and minimize carrier wash out from the tumor and upon triggering free drug become available in close to its targets. Upon equipping targeted triggered nanocarriers with imaging agents, visualization of metastatic loci and subsequent triggering drug release can eradicate micrometastases.

*Azadeh Haeri is currently working as Assistant Professor of Department of Pharmaceutics, School of Pharmacy, Shahid Beheshti University of Medical Sciences, Tehran, Iran. She could be reached at the following e-mail address: a_haeri@sbmu.ac.ir.*
